# Endométriose de la paroi abdominale: à propos de deux cas

**DOI:** 10.11604/pamj.2022.42.54.32449

**Published:** 2022-05-18

**Authors:** Mohamed Hedfi, Nada Essid, Ferdaous Trabelsi, Hakim Znaidi

**Affiliations:** 1 Service de Chirurgie Générale, Hôpital Régional de Zaghouan, Zaghouan, Tunisie,; 2 Service de Gynéco-Obstétrique, Hôpital Régional de Zaghouan, Zaghouan, Tunisie

**Keywords:** Paroi abdominale, endometriose, chirurgie, cas clinique, Abdominal wall, endometriosis, surgery, case report

## Abstract

L'endométriose de la paroi abdominale est une maladie rare qui se développe généralement sur une cicatrice de césarienne. Bien que fréquemment observée dans le tissu adipeux cutané et sous-cutané en regard de la cicatrice de césarienne, sa localisation intramusculaire est possible mais reste rare. Le traitement repose sur l'exérèse chirurgicale de la lésion associée ou non à une thérapie hormonale. L´exérèse chirurgicale large reste le traitement de choix de la maladie mais expose au risque de hernie de la paroi abdominale. Nous rapportons deux cas d'endométriose pariétale survenant après cicatrice de Pfannstiel pour césarienne colligés au service de chirurgie de l'Hôpital régional de Zaghouan.

## Abstract (en)

Abdominal wall endometriosis is a rare disease that usually develops in association with a cesarean section scar. Although frequently identified in the skin and subcutaneous adipose tissue adjacent to the cesarean scar, intramuscular localization is possible but rare. Treatment is based on surgical excision of the lesion associated with or without hormone therapy. Wide surgical excision is the treatment of choice, but it exposes to the risk of abdominal wall hernia. We here report two cases of parietal endometriosis occurring after Pfannstiel scar for cesarean section whose data were collected at the Department of surgery in the Zaghouan Regional Hospital.

## Introduction

L´endométriose est définie par l´implantation anormale d´épithélium endométrial fonctionnel avec stroma en dehors de la cavité utérine. Elle touche les femmes en période d´activité génitale avec une prévalence de 10%. Ses principales localisations sont endopelviennes intéressant principalement les organes génitaux internes (ovaires, trompes, myomètre) les ligaments utero sacrés, le ligament large, la vessie. La localisation dans les organes extra génitaux est moins décrite. L´endométriose pariétale est une entité clinique rare; sa survenue sur les cicatrices opératoires post opératoires notamment sur cicatrice de Pfannstiel est d´autant moins rare (0.03 à 0.4% de l´ensemble des endométrioses) mais constitue la localisation pariétale la plus fréquente [1]. Elle est suspectée sur un faisceau d´argument clinique radiologique; mais sa confirmation reste histologique. L´endométriose pariétale révèle d´un traitement chirurgical. Nous rapportons deux observations d´endométriose pariétale survenant sur cicatrice de Pfannstiel colligées au service de chirurgie de l´Hôpital régional de Zaghouan.

## Patient et observation

### Observation 1

**Informations sur la patiente:** il s´agit d´une patiente âgée de 32 ans, sans antécédents notables; G2P2, elle a eu deux accouchements par voie césarienne dont la dernière remonte à 2019, non connue porteuse d´endométriose génitopélvienne; qui a consulté pour une tuméfaction douloureuse au niveau de la cicatrice de Pfannstiel, évoluant depuis 6 mois (soit un délai de 10 mois suivant l´accouchement). L´anamnèse a trouvé un caractère cataménial de la symptomatologie: exacerbation des douleurs pendant les menstruations, sans notion de dysménorrhée, de dysurie ni de dyspareunie. Par ailleurs la patiente rapporte l´augmentation du volume de la masse d´une façon cyclique pendant les menstruations.

**Données de l´examen clinique:** l´examen clinique a objectivé une masse ovalaire faisant 3 cm de grand axe au niveau de l´angle gauche de la cicatrice de Pfannstiel, mal limitée, peu mobile, et sensible. Les touchers pelviens étaient sans anomalies.

**Figure 1:**
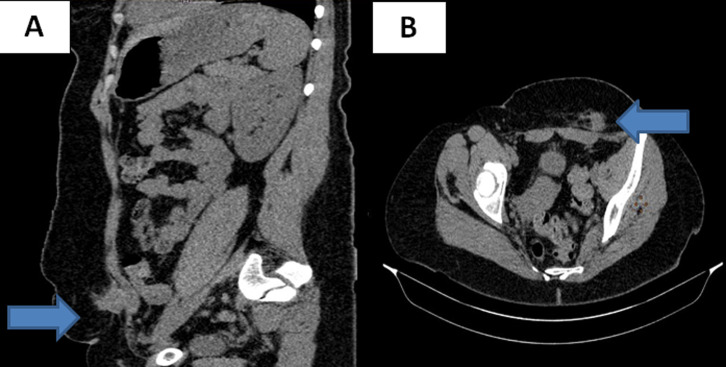
tomodensitométrie abdominopelvienne lésion pariétale au dépend du muscle grand droit: (A) coupe sagittale, B) coupe axiale

**Démarche diagnostique:** l´échographie des parties molles a trouvé une lésion de 18 mm hétérogène hypoéchogène, située en regard du versant gauche de la cicatrice pelvienne La tomodensitométrie (TDM) abdominopelvienne a retrouvé une masse oblongue de 3cm, adhérente au muscle grand droit gauche et à son aponévrose, hypodense et mal limitée (Figure 1A, Figure 1B). Des biopsies n´ont pas été faites. Le diagnostic d´endométriose a été fortement évoqué devant ce faisceau d´arguments anamnestiques, cliniques et para cliniques et la décision était d´opérer la patiente.

**Le traitement chirurgical:** la patiente a été opérée par une reprise sur l´ancienne cicatrice, l´exploration avait trouvé un nodule de 3 cm incrusté dans le muscle grand droit eu une exérèse large de la masse pariétale en emportant la partie du muscle grand droit et son aponévrose en regard de la lésion, avec une réparation de la déhiscence pariétale par une périnéorraphie simple.

**Diagnostic final:** l´étude anatomopathologique a confirmé le diagnostic d´endométriose pariétale en montrant un foyer d´endométriose dans la paroi abdominale.

**Suivi et évolution:** les suites post opératoires immédiates étaient simples. Le suivi avec un recul de 6 mois n´a pas détecté de récidives.

### Observation 2

**Informations sur la patiente:** patiente âgée de 32 ans, aux antécédents d´appendicectomie par voie de Mac Burney à l´âge de 16 ans, G2P2; 2 accouchements par voie césarienne (pour utérus cloisonné) en 2014 et 2017, qui a présenté 4 ans avant son admission, un nodule douloureux au niveau de l´angle gauche de la cicatrice de Pfannenstiel: elle a rapporté un fond douloureux chronique avec une exacerbation cyclique concomitante aux menstruations, par ailleurs elle ne rapporte pas des douleurs similaires en rapport avec la cicatrice de Mac Burney. L´interrogatoire retrouve des dysménorrhées depuis le deuxième accouchement. Depuis quelques mois avant l´admission la patiente rapporte une modification de la coloration de la peau en regard du nodule, devenant bleuâtre pendant les menstruations.

**Données de l´examen clinique:** l´examen avait trouvé un nodule de 2cm au niveau de l´angle gauche de la cicatrice de Pfannenstiel, la cicatrice de Mac Burney était solide et sans anomalies.

**Figure 2:**
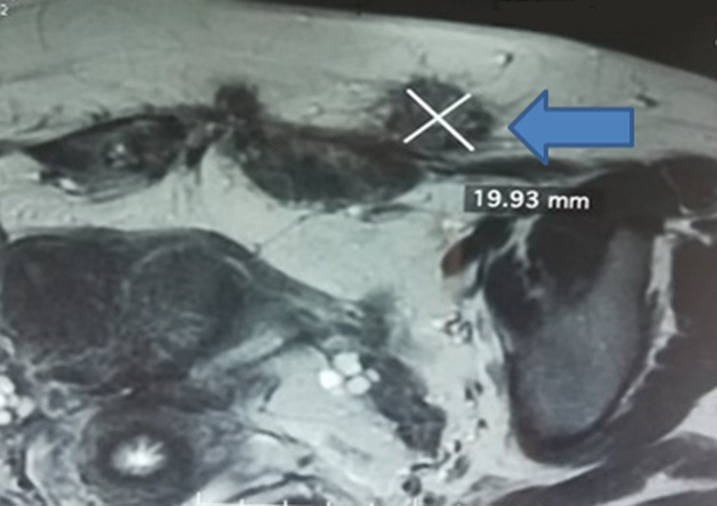
coupe coronale d´une IRM pelvienne en pondération T1 séquence saturation de graisse (fat sat); nodule paramédian gauche siégeant sur la cicatrice de césarienne mesurant 17x19 mm

**Démarche diagnostique:** la TDM abdominale avait trouvé deux nodules de 3x2 cm et de 1x2 cm au niveau des muscle grands droits de part et d´autre en regard de la cicatrice de Pfannenstiel, de même densité avant et après injection de produit de contraste que celle de l´utérus évoquant des lésions d´endométriose. Une Imagerie à Résonance Magnétique (IRM) pelvienne a été réalisée pour mieux identifier les lésions et à la recherche de lésions d´endométriose pelvienne profonde justifiant un éventuel traitement médical. L´IRM pelvienne avait montré un utérus cloisonné, avec présence de multiples nodules endométriosiques sur l´ensemble de la cicatrice opératoire de la césarienne, dont un mesurant 17x 19 mm enchâssés dans la graisse sous cutanée et dans l´épaisseur des muscles droits droit et gauche (Figure 2). Par ailleurs, absence de lésions en faveur d´endométriose pelvienne profonde.

**Figure 3:**
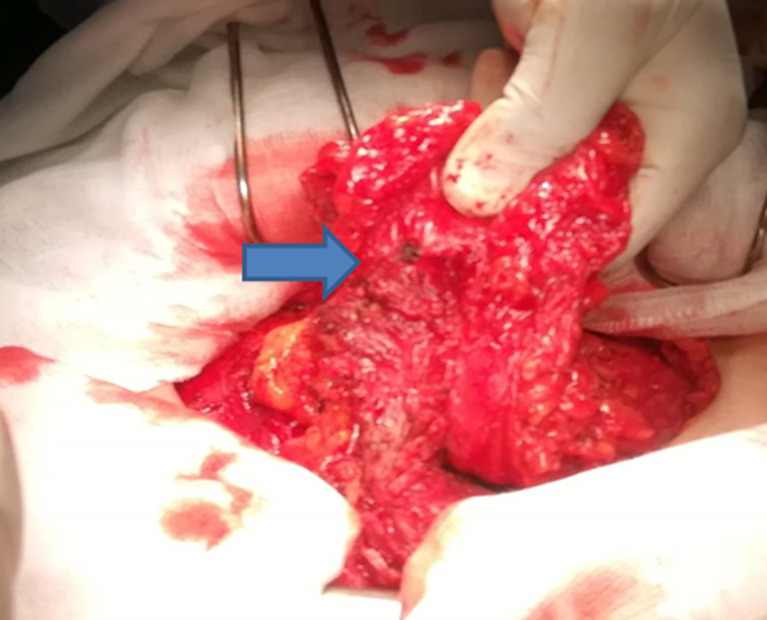
vue peropératoire, nodule d’endométriose sur le muscle grand droit

**Figure 4:**
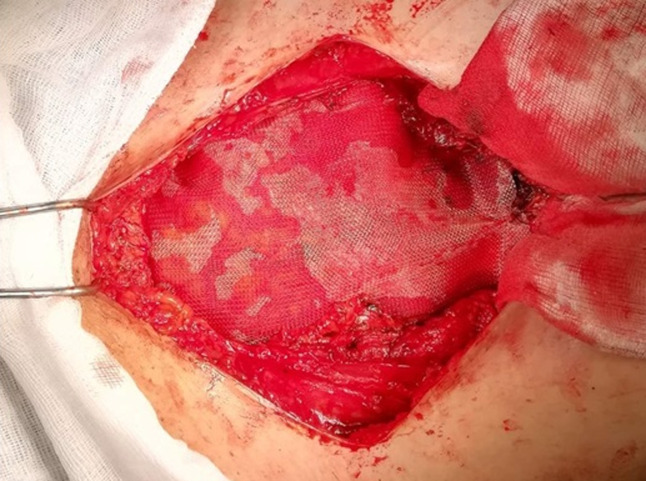
vue peropératoire, réparation pariétale par une plaque retro-musculaire préfaciale

**Le traitement chirurgical:** la patiente a été opérée en reprenant l´ancienne cicatrice de Pfannenstiel, on retrouve un nodule ayant des rapports avec l´aponévrose du muscle grand droit gauche, et un autre nodule sous aponévrotique ayant des rapports avec les muscles grands droits à droite et s´étendant jusqu´au pubis (Figure 3). Une excision musculaire et aponévrotique large emportant les nodules a été effectuée en passant par du tissu sain réalisant une résection en monobloc. Devant l´importance de la perte de substance sur la paroi abdominale exposant au risque de hernie, une pariétoplastie a été réalisée en interposant une plaque de polypropylène en rétro musculaire préfascial (Figure 4).

**Figure 5:**
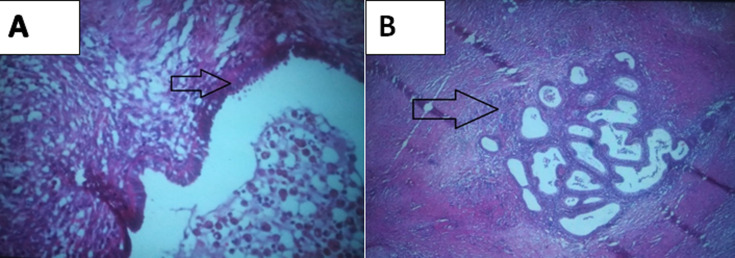
aspect histologique (HEx40), A) présence de glandes bordées par un épithélium pseudo stratifié avec présence de chorion cytogène; B) foyer d´endométriose au sein de la paroi abdominale

**Diagnostic final:** l´examen anatomopathologique macroscopique révèle 2 pièces graisseuses présentant à la coupe de très nombreux foyers hémorragiques. L´étude histologique a confirmé le diagnostic d´endométriose pariétale avec présence de glandes bordées par un épithélium pseudo stratifié avec présence de chorion cytogène (Figure 5A, Figure 5B).

**Suivi et évolution:** les suites opératoires immédiates étaient simples. Le suivi avec un recul de 6 mois n´a pas détecté de récidives.

## Discussion

L´endométriose est définie par l´implantation ectopique de l´épithélium endométrial fonctionnel en dehors de la cavité utérine [1]. C´est une pathologie chronique dont le diagnostic est souvent évoqué tardivement par rapport au début de la symptomatologie du fait d´une méconnaissance de la pathologie. Elle concerne principalement les femmes en âge de procréer avec un pic autour de 40 ans [1]. Les principales localisations sont pelviennes. La localisation pariétale est rare. La localisation sur cicatrice de chirurgie gynécologique ou obstétricale à laquelle on s´intéresse est encore plus rare [1,2]. La physiopathologie de l´endométriose est encore mal élucidée et incertaine, cependant plusieurs théories ont été avancées dont on cite [1,2]. 1). La théorie de l´implantation: décrite en 1927 par Sampson: les cellules endométriosiques provenant du reflux du sang menstruel par les trompes vers la cavité péritonéale s´implantent de façon ectopique. En présence d´un dysfonctionnement du système d´épuration ou en raison de l´abondance du reflux. Les cellules infiltrent le mésothélium des vaisseaux des tissus envahis générant ainsi une angiogenèse. 2) La théorie métaplasique: selon cette théorie, les cellules de l´épithélium cœlomiques (tissu d´origine embryologique présent au niveau du péritoine, la plèvre, le péricarde) subissent une métaplasie en cellules endométriales, cette théorie pourrait expliquer les cas de localisation pleurales et les cas observés chez les hommes sous traitement ostrogénique. 3) La théorie métastatique: elle stipule que la dissémination des cellules endométriales est possible par voie lymphatique et vasculaire.

Dans notre contexte: l´endométriose pariétale peut être expliquée par la théorie d´implantation parce qu´il s´agit d´est une dissémination iatrogène et involontaire des cellules endométriales au cours d´une chirurgie abdominopelvienne. Plusieurs facteurs de risque ont été incriminés dans la genèse de l´endométriose dont on cite l´activité génitale et toute exposition prolongée aux œstrogènes (puberté précoce, ménopause tardive) mais aussi des intervalles prolongés entre les grossesses. Les troubles menstruels ont été aussi incriminés: les femmes endométriosiques ont généralement des cycles courts avec un flux abondant et prolongé, ce qui pourrait correspondre à une exposition à un reflux menstruel abondant et plus fréquent. Les anomalies congénitales du tractus génital sont aussi considérées comme facteur de risque. En effet tout obstacle à l´écoulement normal du flux menstruel favorise potentiellement le reflux. En ce qui concerne nos deux patientes, non connues porteuses d´endométriose génitopelvienne, l´âge de diagnostic est de 32 ans chez les 2 patientes. L´âge de la ménarche était à l´âge de 14 et 12 ans. Les cycles sont réguliers de 28 jours sans anomalies du flux chez les deux patientes. Une malformation congénitale est présente chez l´une des patiente (utérus cloisonné). Sur le plan clinique: la symptomatologie de l´endométriose pariétale est essentiellement d´une masse douloureuse avec un caractère cataménial. Ce caractère cyclique de la douleur est un élément important d´orientation mais non indispensable pour évoquer le diagnostic. Ces signes peuvent apparaitre dans les semaines voire des années suivant l´intervention. Chez notre patiente, l´intervalle entre l´intervention et le début de la symptomatologie est de 16 mois pour une patiente et de 12 mois pour l´autre [1,2]. Les lésions siégeant sur les cicatrices opératoires classiquement en regard de la cicatrice de Pfannenstiel sont en général des masses faisant en moyenne 2 cm douloureuses. Il a été aussi décrit un changement cyclique du comportement des lésions très superficielles, à type de saignement et de fistulisation à la peau [1].

Chez l´une des patientes, un changement de la coloration de la peau en regard de la masse pendant les menstruations a été noté. La localisation pariétale chez nos patientes était sur la cicatrice de Pfannenstiel. Il est à noter que la cicatrice de Mac Burney de la 2^e^ patiente a été indemne d´endométriome. Les images échographiques et scannographies des endométrioses cicatricielles sont peu spécifiques. Il s´agit soit d´aspect de collection liquidienne soit d´aspect tissulaire. En effet l´aspect kystique/tissulaire de la lésion dépend de l´imprégnation hormonale en fonction du cycle. La composante liquidienne a été trouvée à l´échographie chez la 1^ère^ patiente [3]. L´intérêt de la TDM réside dans l´étude de l´envahissement en profondeur des lésions. L´IRM est l´examen de choix à réaliser; à la recherche de lésions endométriosiques profondes indiquant un traitement médical associé à la chirurgie d´exérèse [1,3]. Chez l´une de nos patientes, le diagnostic a été suggéré par la TDM et consolidé par l´IRM. Le taux sérique de CA125 peut être augmenté en corrélation avec la prolifération épithéliale des lésions endométriosiques. Chez notre patiente les marques tumorales n´ont pas été demandés. La confirmation du diagnostic reste histologique montrant la présence, sur les pièces opératoires de glandes endométriales en position ectopique avec des tubes glandulaires, du chorion cytogène et des fibres musculaires.

Le traitement de référence des endométrioses pariétales est l´exérèse chirurgicale large d´emblée quitte à réaliser une pariétoplastie pour combler la déhiscence aponévrotique [2,4]. En ce qui concerne notre étude l´une des patientes a eu une exérèse isolée, l´autre a eu une exérèse avec recours à une pariétoplastie par plaque. Bien qu´aucun bénéfice n´a été démontré; il est conseillé en cas de laparotomie de réaliser un lavage abondant de la cicatrice et de changer de gants avant sa fermeture. En cas de cœlioscopie, pour prévenir la greffe endométriosique sur les orifices des trocarts, il révèle de la bonne pratique chirurgicale d´extraire les pièces opératoires dans un sac de protection de façon systémique [1,3,4].

## Conclusion

Les endométriomes pariétaux sont rares. La localisation sur cicatrice de césarienne est la plus fréquente. Le diagnostic peut être difficile mais doit être évoqué devant une tumeur pariétale douloureuse et cyclique sur cicatrice gynécologique ou obstétricale. Les examens radiologiques servent à caractériser la lésion (nombre, siège, dimensions, profondeur) et à rechercher d´éventuels signes de maladie profonde indiquant un traitement médical après exérèse les lésions superficielles. La chirurgie doit être large pour éviter les récidives. Le diagnostic n´est confirmé que par l´étude anatomopathologique. Malgré que l´endométriose pariétale obéit à la théorie d´implantation iatrogène accidentelle lors de la manipulation des plaies en per opératoire, aucune efficacité des mesures préventives per opératoires n´ont été encore démontrées.
